# How to Improve the Cognitive Health of Middle-Aged and Elderly People: Evidence From China Family Panel Studies

**DOI:** 10.3389/fpubh.2022.799255

**Published:** 2022-02-04

**Authors:** Wei Yuan, Min Jiang, Shuying Gong

**Affiliations:** ^1^School of Business Administration, Hubei University of Economics, Wuhan, China; ^2^Shanghai Institute of Tourism, Shanghai Normal University, Shanghai, China; ^3^College of Business, Shanghai University of Finance and Economics, Shanghai, China

**Keywords:** internet use, internet involvement, cognitive health, satisfaction of emotional attachment with children, heterogeneity

## Abstract

In the context of the rapid development of the internet and the increasing severity of the aging problem, and in order to promote aged health and help construct a healthy society. We use micro survey data from the 2016 China Family Panel Studies (CFPS) to investigate the impact of the internet on the cognitive health of middle-aged and elderly people (those aged 40 and above). The robust results suggest that the internet plays a significant positive role in the cognitive health of middle-aged and elderly people in terms of internet use and internet involvement. This effect does not change significantly with differences in gender, household registration, location, or household composition, but there are heterogeneity effects due to differences in education. Further analysis indicates that the satisfaction of emotional attachment with children is the internal mechanism of the internet's influence on the cognitive health of people in this age group. Our paper both help scholars and the public to better understand the impact of the internet on the cognitive health of middle-aged and elderly people and clarifies different methods of defining the internet.

## Introduction

The aging of the population has become a global concern. As one of the countries that bears the brunt of this issue, China has a demographic situation that is not promising. As of 2020, the total number of elderly people in China exceeded 250 million, accounting for about 20% of the population.^1^ Faced with the reality of this deepening trend, China has paid great attention to healthy aging strategies (1). The cognitive health of elderly people is a very important part of healthy aging (2). Data show that there are more than 24 million dementia patients in the world, and Chinese patients with dementia account for about a quarter of cases globally, with an average of 300,000 new cases every year.^2^ It is clear that the cognitive health status of the elderly in China is worrying, and dealing with this problem has become a top priority for the whole country. Therefore, determining which factors are beneficial to the cognitive health of the elderly will not only enrich existing theoretical results but also contribute to the realization of healthy aging, with important practical implications for society.

In 2015, Chinese Premier Li proposed a policy guide on the Internet Plus initiative, which further boosted the rapid development of the internet. More and more elderly people have since become internet users. In 2017, the proportion of elderly people in China's total population using the internet was 4.8%. The per capita time spent on the internet by the elderly increased from 98 hours in December 2017 to 118 hours in December 2018, a year-on-year increase of 20.7%.^3^ Many studies have focused on the relationship between the internet and the health of elderly people (3–6). However, these studies mainly investigate the value of the internet for the physical and mental health of the elderly, seldom paying attention to its influence on their cognitive health or the mechanism of that influence.

Building on existing research, we explore the relationship between the internet and the cognitive health of middle-aged and elderly people in the context of the aging population and the Internet Plus initiative. In terms of research objects, we include middle-aged as well as elderly people, since the middle-aged group is in a transitional stage in relation to old age and is also the group that is most often ignored by society. With the increasing aging of the population, more attention should be paid to the cognitive health of the middle-aged group in the transition period.

In order to explore the effect of the internet on the cognitive health of middle-aged and elderly people, we use two variables-internet use (dummy variable) and internet involvement (continuous variable) to measure the internet usage. We use a series of robustness tests, including instrumental variables and propensity score matching, to overcome potential endogeneity and other problems and to ensure the accuracy and reliability of the results. We also analyze the heterogeneity of the effects of the internet on the cognitive health of different groups of middle-aged and elderly people. Finally, drawing on the typical dependent self-construction of Chinese people and the perspective of family intergenerational relationships, we conduct further analysis to determine whether the satisfaction of emotional attachment with children is the psychological mechanism by which the internet affects the cognitive health of middle-aged and elderly people.

Through a series of theoretical and empirical analyses, we reach the following conclusions. Internet use positively affects the cognitive health of middle-aged and elderly people, as does internet involvement. In general, the internet (internet use and internet involvement) has a significant positive impact on the cognitive health of people in that age group. These effects do not change with differences in gender, household registration, location, or household composition. However, there is heterogeneity in relation to different levels of education; the main effect is found in middle-aged and elderly people who received a general education but not in those who are illiterate or received a higher education. Further analysis indicates that the satisfaction of emotional attachment with children is the mechanism of the relationship between the internet and the cognitive health of middle-aged and elderly people.

Our paper makes several contributions. First, we examine the casual effect of the internet (internet use and internet involvement) and cognitive health of middle-aged and elderly people and prove that its effects are beneficial. This not only echoes the dual context of China's Internet Plus initiative and the aging of the population but also helps to expand the scope of research on factors that influence cognitive health, providing new perspectives for future work.

Second, our heterogeneity analysis reveals that the effect of the internet on the cognitive health of middle-aged and elderly people cuts across distinctions in gender, household registration, and geographical location. Therefore, the internet can be used to help deal with health inequalities or deterioration in cognitive health caused by aging, although it should be noted that for those who received a higher education need to be explored.

Third, the satisfaction of emotional attachment with children plays a significant mediating role in the impact of the main effect. Thus, on the one hand, our research conclusions are useful for researchers and the public to understand and recognize the relationship between the internet and the cognitive health of middle-aged and elderly people. On the other hand, they provide new empirical evidence for the Chinese self-construction in terms of a dependent and prominent family intergenerational relationship from a micro perspective, which enriches the theory of self-construction and family intergenerational relations.

Fourth, our study is innovative in subdividing the dependent internet variable into two levels: internet use and internet involvement. This approach both helps scholars and the public to better understand the impact of the internet on the cognitive health of middle-aged and elderly people and clarifies methods of defining the internet.

Fifth, our paper reveals the impact of the internet on the cognitive health of middle-aged and elderly people and its mechanism from an instrumental perspective. This lays the foundations for future research to explore the issue from a labeling perspective (e.g., whether middle-aged and elderly people are marginalized or left behind in the internet age).

## Theory and Hypotheses

The internet has quietly and successfully penetrated all aspects of life. Numerous studies have consistently shown its significant impact on the real economy, industrial manufacturing, financial development, technological innovation, transportation, and other fields (7, 8), as well as its contribution to significant changes in residents' health, economic decision-making, payment methods, leisure and entertainment, and social interactions (9, 10). Although scholars have conducted research on the relationship between the internet and the health of residents, gaps remain. First, in terms of research objects, little attention has been paid to the relationship between the internet and the health of middle-aged people. As mentioned above, the middle-aged group represents a transitional stage in terms of age, and it is also the group that is most often neglected by society. With the aging of the population, more attention should be paid to the health of middle-aged people in that transition period. Second, in terms of research scope, previous studies have focused mainly on the relationship between the internet and physical/mental health, ignoring its relationship with cognitive health, which is the general and basic cognitive ability possessed by an individual (11, 12). Scholars have explored the impact of physical exercise, taking care of grandchildren, lifestyle, social interactions, intergenerational support, and other variables on the cognitive health of the elderly (13, 14), but few have investigated the impact of the internet on their cognitive health.

In order to broaden the existing theoretical results, and at the same time to reflect the background of the aging population and the Internet Plus initiative, our study takes middle-aged and elderly people as its research object and explores the relationship between the internet and the cognitive health of people in those groups. The internet has the following primary functions: helping people to obtain and transmit information, and helping them to achieve communication across time and space. Our paper will focus on both functions to develop a number of theoretical constructions.

First, from the perspective of the first function, Small et al. (15) performed functional magnetic resonance imaging (MRI) on middle-aged and elderly people. They found that when middle-aged and elderly people search on the internet, their brain activity relates to the control of decision-making and complex reasoning; the signal strength of the related areas of the brain increases significantly. This indicates that the internet searching experiences of middle-aged and elderly people may change the ability of their brains to respond to the neural circuits that control decision-making and complex reasoning, and that changes in brain response ability can help enhance individual cognitive health (16). Therefore, the internet may improve the cognitive health of middle-aged and elderly people by changing the brain's ability to respond. Moreover, middle-aged and elderly people use the internet to access massive amounts of new knowledge, and to learn and master new skills (17). This not only enriches their daily lives and spiritual world, thereby increasing their life satisfaction, but also helps them to gain timely insight into social changes, helping them to keep pace with society and improving their self-confidence. Without these opportunities, middle-aged and elderly people are at risk of being marginalized by younger people. Life satisfaction and self-confidence are both more likely to lead to positive emotions (18) and, according to the expansion-construction theory of positive emotions, positive emotions cause richer thoughts, increase cognitive flexibility, expand attention spans, and promote cognitive map innovation (19). Therefore, the internet can enhance the cognitive health of middle-aged and elderly people by improving their life satisfaction and self-confidence.

From the perspective of the second function, middle-aged and elderly people not only use the internet to communicate with families and friends (20) but also to meet strangers. Such online relations can lead to offline social activities and, ultimately, more in-depth communication. Among middle-aged and elderly people, communication can create closer interpersonal relationships, reduce loneliness, and improve levels of happiness (17, 21). Both a decrease in loneliness and an increase in happiness immerse middle-aged and elderly people in the positive emotions (18) that are beneficial to cognitive health (19). Therefore, the internet can improve the cognitive health of middle-aged and elderly people by reducing loneliness and increasing happiness.

In conclusion, from both perspectives, the internet has a positive effect on the cognitive health of middle-aged and elderly people. Accordingly, we propose the following hypotheses:

*H1: Internet use is significantly positively correlated with the cognitive health of middle-aged and elderly people; that is, compared to middle-aged and elderly people who do not use the internet, those who use the internet have better cognitive health*.
*H2: Internet involvement is significantly positively correlated with the cognitive health of middle-aged and elderly people; that is, compared with middle-aged and elderly people who are less involved in the internet, those who are highly involved in the internet have better cognitive health*.

## Methods

### Data

The data used in the empirical analysis come from the 2016 China Family Panel Studies (CFPS).^4^ The data focuses on the economic and non-economic welfare of Chinese residents, as well as many related research topics including economic activities, educational achievements, family relations and family dynamics, population migration, health problems, etc. It is a national, large-scale and multidisciplinary social tracking survey project. The CFPS sample covers 25 provinces / cities / autonomous regions, the target sample size contains more than 16,000 households, and the interviewees include all family members in the sample households^5^ We followed previous studies (22) in sorting the samples by (1) matching the family and adult databases, (2) deleting samples for respondents under 40 years of age and (3) deleting samples with missing important variables. There is no consensus in the literature on the age range of middle-aged and elderly people. Therefore, in order to avoid the problem of age range definitions affecting the research results, we changed the sample age ranges in the robustness tests. (4) we also adjusted the important variables to a significance level of 1% *Winsorize* to avoid abnormal values that might affect the results. This process yielded 12,000 valid samples.

### Variables

#### Dependent Variable

To define cognitive health, we used Zhou et al.'s (23) item, “Can you remember the main things that happened to you in a recent week?” (1 = can only remember a little, 2 = can only remember a few, 3 = can remember half, 4 = can remember most, and 5 = can fully remember). To avoid bias from the choice of measurement methods, in the robustness tests we used different measurements of cognitive health.

#### Independent Variables

In order to fully reveal the impact of the internet on the cognitive health of middle-aged and elderly people, we subdivided the independent variable into two levels: internet use (dummy variable) and internet involvement (continuous variable). To measure internet use, we took items from the CFPS (2016) questionnaire: “Do you use mobile devices to surf the internet, such as a mobile phone or a tablet?” and “Do you use a laptop online?” (1 = yes, 0 = no). If the respondent used a mobile phone, a tablet, or a laptop to surf the internet, he/she was regarded as a person who uses the internet and was assigned the value 1; otherwise, he/she was regarded as a person who does not use the internet and was assigned the value 0. To define internet involvement, we again used an item from the CFPS (2016) questionnaire: “How often do you use the internet for learning in general (such as searching learning materials, taking online learning courses, et al.)?” (1 = never, 2 = once every few months, 3 = once a month, 4 = two or three times a month, 5 = once or twice a week, 6 = three or four times a week, 7 = almost every day). We also used the following items (with the same coding): “In general, how often do you use the internet to work?”; “In general, how often do you use the internet for social activities (such as chatting in WeChat or posting photos in Weibo)?”; “In general, how often do you use the internet for entertainment (such as watching videos or downloading songs)?”; and “In general, how often do you use the internet for commercial activities (such as online banking or online shopping)?” We measured the average values for these five items; the greater the value, the higher the degree of involvement with the internet.

#### Control Variables

In accordance with previous studies, we controlled for the following factors that may affect the cognitive health of middle-aged and elderly people: individual characteristic variables (age, gender, education level, marital status, political status, household registration, and whether exercise is taken); family characteristic variables (per capita household income); and city characteristic variables (provincial GDP, living in a municipality area, living in the eastern area, or living in the central area).

Table 1 presents the descriptive statistics for the variables. The average score for cognitive health of middle-aged and elderly people in China is 2.922, which indicates that our sample is representative. Nevertheless, this also suggests that the cognitive health of middle-aged and elderly people is now a serious issue that deserves widespread attention from the society.

**Table 1:** Descriptive statistics.

**Variable**	**Observations**	**Mean**	**S.D**.	**Min**.	**Max**.
Dependent variable					
Cognitive health	12,685	2.922	1.284	1	5
Independent variables					
Internet use	12,702	0.104	0.305	0	1
Internet involvement	12,699	1.222	0.771	1	7
Control variables					
Gender (Male = 1)	14,155	0.489	0.500	0	1
Age	14,167	52.549	9.220	40	88
Years of education	13,612	4.774	4.796	0	19
Marital status (Married = 1)	35,606	0.995	0.072	0	1
Political status (Yes = 1)	14,157	0.105	0.307	0	1
Nonagricultural population (Yes = 1)	29,733	0.254	0.436	0	1
Smoking (Yes = 1)	12,689	0.765	1.260	0	3.714
Exercise (Yes = 1)	35,606	0.801	0.400	0	1
Break time (Yes = 1)	12,701	0.569	0.495	0	1
Medicare insurance (Yes = 1)	14,137	0.932	0.252	0	1
Log of per capita income	14,127	9.331	1.005	6.727	11.918
Provincial (urban) level GDP	14,316	10.112	0.669	6.935	11.196
Municipality (Yes = 1)	14,316	0.112	0.316	0	1
Eastern area (Yes = 1)	14,316	0.472	0.499	0	1
Central area (Yes = 1)	14,316	0.290	0.454	0	1

*In the robustness tests, we also control variables such as whether the household uses tap water, the local level of medical care, and the local environmental quality. Due to space limitations, we do not report the results for these variables, which can be obtained from the authors on request*.

**Figure 1:**
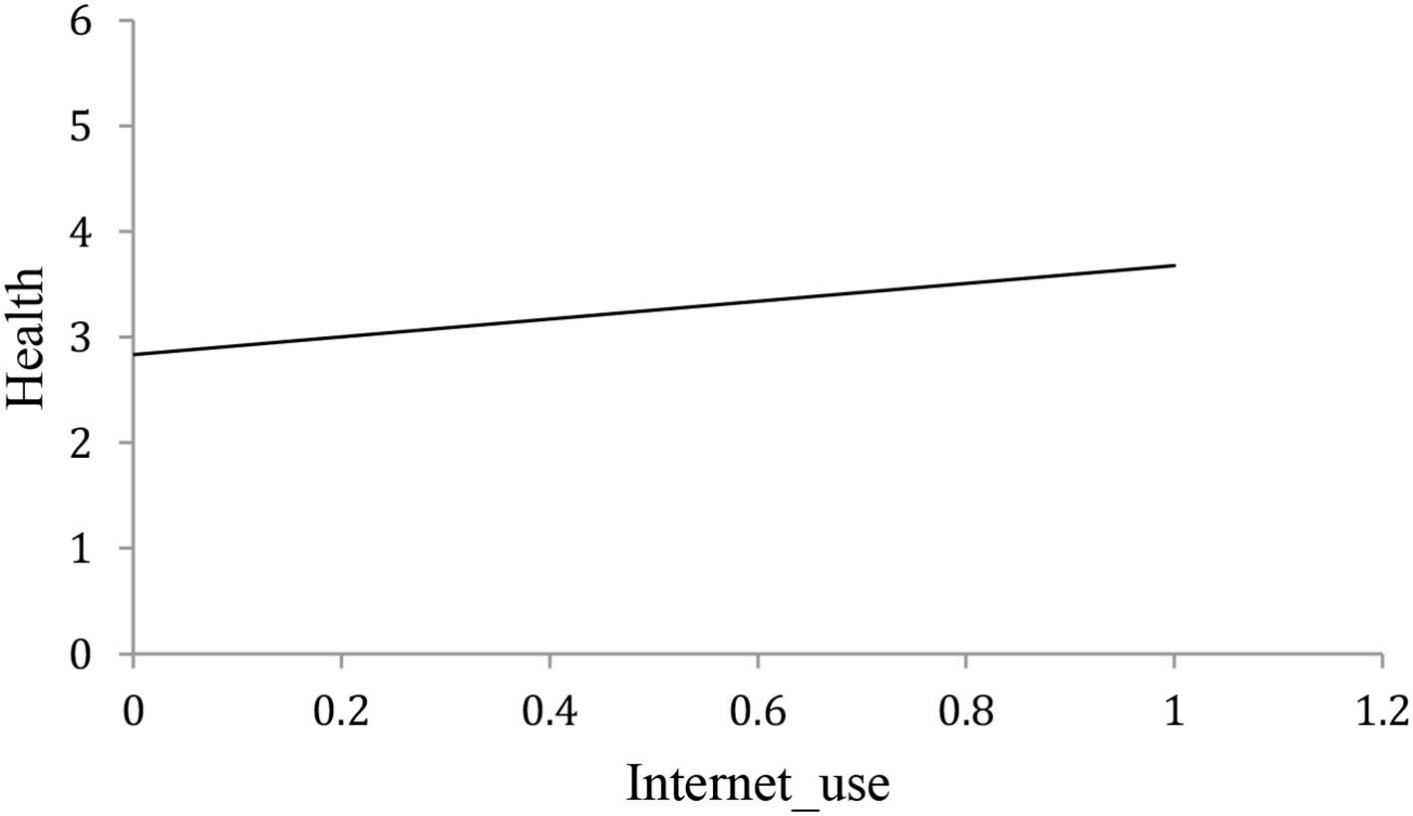
Relationship between internet use and cognitive health of middle-aged and elderly people (horizontal axis shows internet use, vertical axis shows cognitive health).

**Figure 2:**
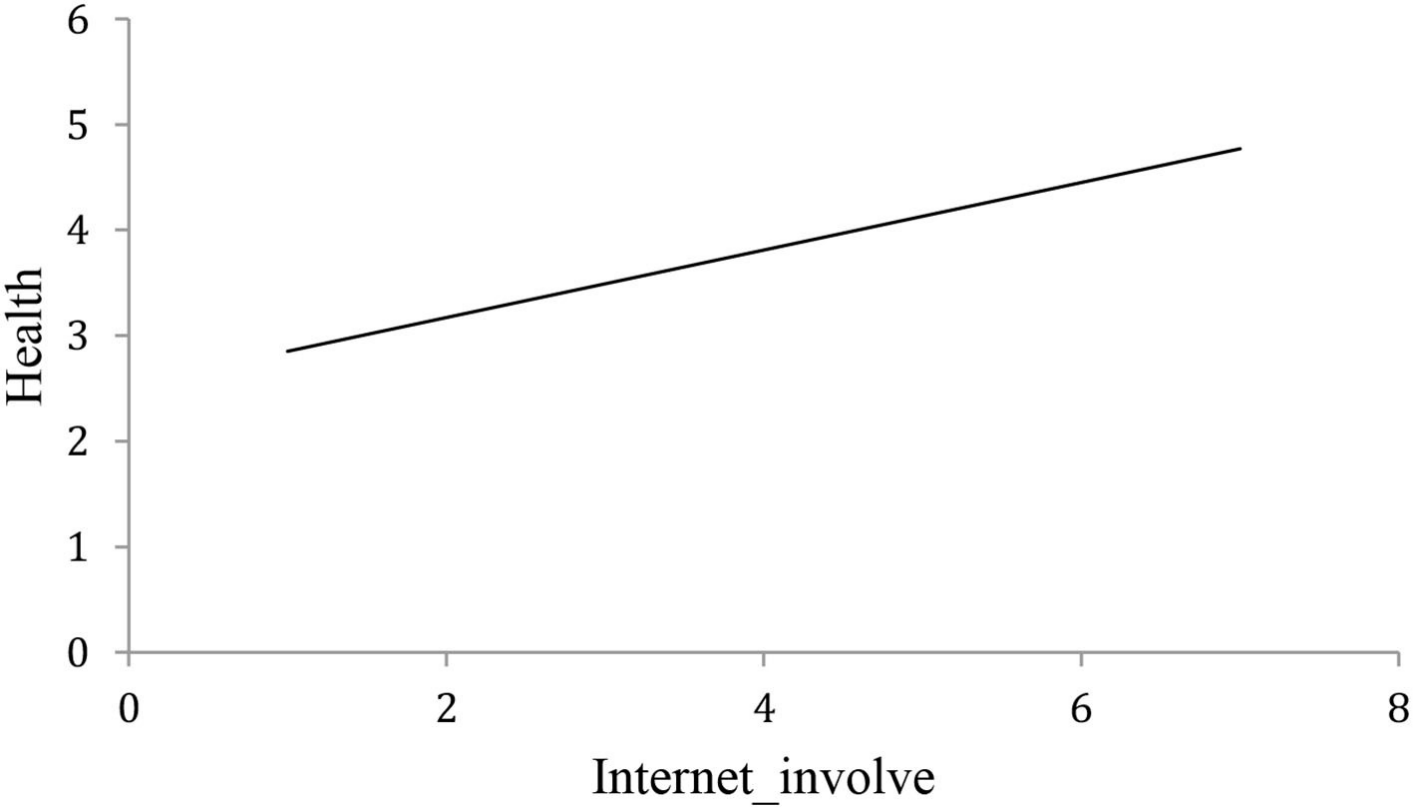
Relationship between internet involvement and cognitive health of middle-aged and elderly people (horizontal axis shows internet involvement, vertical axis shows cognitive health).

In order to examine the relationship between the internet and the cognitive health of middle-aged and elderly people, we used scatterplots (Figures 1, 2). These give a preliminary indication that internet use has a significant positive correlation with the cognitive health of middle-aged and elderly people, as does internet involvement. These results provide initial support for the view that the internet is beneficial to the cognitive health of middle-aged and elderly people, and we seek further verification in what follows.

### The Model

To verify the impact of the internet (internet use and internet involvement) on the cognitive health of middle-aged and elderly people, we controlled individual characteristics, family characteristics, and the characteristics of the city area, as shown in Models (1) and (2):


(1) $$\begin{array}{l} Health_{i}=\beta_{0}+\beta_{1}{Internet\text{\_}use}_{i}+\Gamma X_{i}+\varepsilon_{i} \end{array}$$

(2) $$\begin{array}{l} Health_{i}=\beta_{0}+\beta_{1}{Internet\text{\_}involve}_{i}+\Gamma X_{i}+\varepsilon_{i} \end{array}$$

The above models are both Order-Probit models with the dependent variable *Health*_*i*_, which represents the cognitive health status of individual *i*. In Model (1), the independent variable is *Internet*_*use*_*i*_ (dummy variable), which represents the individuals' internet use situation (use = 1). In Model (2), the independent variable is *Internet*_*involve*_*i*_ (continuous variable), which represents the degree of the individual's internet involvement. The coefficient β_1_ in each model captures the effect of the internet on cognitive health, and *X*_*i*_ represents a series of control variables that may affect the cognitive health of middle-aged and elderlypeople.

## Empirical Results

### Main Effect Results

Before the empirical analysis, to ensure the validity and consistency of the model specification, we conduct a test for multicollinearity. The two models' mean scores of the variance inflation factor (VIF) are 1.510. Since all the variables' VIF scores do not exceed the threshold of 10, thus, multicollinearity is not problematic (24). Table 2 reports the regression results for the effect of the internet on the cognitive health of middle-aged and elderly people. Models (1) and (4) are the basic models at different levels, and both contain only the core independent variables; in Models (2) and (5), we add control variables (not including the urban dummy variables) on the basis of the basic model; Models (3) and (6) are full models at different levels, each with significant robustness results.

**Table 2:** Model results for the effect of the internet on the cognitive health of middle-aged and elderly people.

**Model/Variable**	**(1)**	**(2)**	**(3)**	**(4)**	**(5)**	**(6)**
Internet use	0.693***	0.183***	0.173***			
(Use = 1)	(0.031)	(0.035)	(0.035)			
Internet				0.265***	0.074***	0.071***
involvement				(0.012)	(0.014)	(0.014)
Age		−0.009***	−0.009***		−0.009***	−0.010***
		(0.001)	(0.001)		(0.001)	(0.001)
Gender		0.201***	0.198**		0.202***	0.200***
(Male = 1)		(0.024)	(0.024)		(0.024)	(0.024)
Years of		0.034***	0.035***		0.034***	0.035***
education		(0.002)	(0.002)		(0.002)	(0.002)
Marital status		0.191**	0.182**		0.190**	0.181**
(Married = 1)		(0.088)	(0.088)		(0.088)	(0.088)
Political status		0.104***	0.109***		0.098***	0.104***
(Yes = 1)		(0.033)	(0.033)		(0.033)	(0.033)
Nonagricultural		0.110***	0.096***		0.112***	0.098***
population		(0.025)	(0.026)		(0.025)	(0.026)
Medical		0.056	0.063		0.056	0.063
(Yes = 1)		(0.039)	(0.039)		(0.039)	(0.039)
Per capita		0.169***	0.159***		0.169***	0.159***
income		(0.011)	(0.011)		(0.011)	(0.011)
Exercise		0.105***	0.106***		0.106***	0.107***
(Yes = 1)		(0.020)	(0.020)		(0.020)	(0.020)
Break		−0.025	−0.017		−0.024	−0.017
(Yes = 1)		(0.020)	(0.020)		(0.020)	(0.020)
Smoking		0.028***	0.028***		0.029***	0.029***
		(0.009)	(0.009)		(0.009)	(0.009)
Provincial		0.094***	0.140***		0.095***	0.140***
(urban) GDP		(0.015)	(0.020)		(0.015)	(0.020)
Municipality			0.159***			0.160***
(Yes = 1)			(0.037)			(0.037)
Eastern area			−0.108***			−0.106***
(Yes = 1)			(0.036)			(0.036)
Central area			−0.117***			−0.117***
(Yes = 1)			(0.031)			(0.031)
LR chi^2^	504.83***	1,802.85***	1,834.38***	465.11***	1,802.99***	1,834.72***
R^2^	0.0126	0.0471	0.0479	0.0116	0.0471	0.048
Observations	12,685	12,131	12,131	12,682	12,129	12,129

*The results reported in columns (1)–(6) are the original estimation coefficient, not the marginal effect. The value in parentheses is the standard error.*

*** and ** represent significance at the 1% and 5% levels, respectively.

The results for Model (1) suggest that internet use is positively and significantly correlated with the cognitive health of middle-aged and elderly people (β = 0.693, *p* < 0.01), and this result is still robust in Model (2) (β = 0.183, *p* < 0.01) and in Model (3) (β = 0.173, *p* < 0.01). Therefore, hypothesis H1 is verified; that is, internet use significantly improves the cognitive health of middle-aged and elderly people.

The results for Model (4) suggest that internet involvement is positively and significantly correlated with the cognitive health of middle-aged and elderly people (β = 0.265, *p* < 0.01), and the result is still robust in Model (5) (β = 0.074, *p* < 0.01) and in Model (6) (β = 0.071, *p* < 0.01). Therefore, hypothesis H2 is also verified; that is, involvement with the internet significantly improves the cognitive health of middle-aged and elderly people. In conclusion, the internet has a significantly positive impact on the cognitive health of middle-aged and elderly people, which means that it is beneficial to their cognitive health.

### Robustness Tests

#### Endogeneity Tests

In the previous analysis, we established that the internet significantly affects the cognitive health of middle-aged and elderly people. When using the internet, people need certain cognitive abilities. Accordingly, it can be assumed that middle-aged and elderly people with poor cognitive health (such as those suffering from Alzheimer's disease) are less inclined to use the internet and less involved in the internet because of the impairment of their cognitive ability. Thus, the cognitive health of middle-aged and elderly people has an important influence on whether they use the internet and on their degree of involvement with the internet. Therefore, to avoid endogeneity problems caused by the two-way causal relationship between internet use (internet involvement) and the cognitive health of middle-aged and elderly people, we follow the approach of Czernich et al. (25), taking the widespread rate of fixed phones at the provincial (municipal) level in 1995 as an instrumental variable for internet use and internet involvement. The reasons for this decision are as follows. First, China began to provide civilian internet services in 1996. Since internet access is based on all kinds of Digital Subscriber Lines (xDSL) technology, and the transmission medium of Digital Subscriber Line (DSL) is copper telephone line, the number of fixed phone lines in 1995 has a high correlation with the rate of civilian internet use in 1996 and the future development of the internet. Second, although the widespread rate of fixed phones at the provincial (municipal) level in 1995 determines the availability of a certain communication medium for the spread of the internet, it will not have a direct impact on the cognitive health of middle-aged and elderly people in 2016 (21 years later).

#### Changing the Model Settings

The model settings in previous studies did not control factors such as the environmental and medical conditions of the individual's area. However, these factors may have an important impact on the cognitive health of middle-aged and elderly people, and omitting them may lead to endogeneity problems in the model and bias in the results. In order to eliminate this problem, we followed standard practice in previous research (23), adding factors that may affect the cognitive health of middle-aged and elderly people as additional control variables in the basic model. According to the principle of data availability, we used four variables from the CFPS questionnaire at the provincial (municipal) level to represent environmental factors: air quality, annual number of hours of sunshine, degree of noise pollution, and whether households use tap water for cooking. At the same time, we used the CFPS questionnaire variables for satisfaction with the medical condition of the local area to represent medical factors.

#### Changing the Measurements of Important Variables

In order to avoid the definitions of the variables would affect our findings, we changed the measurement methods for the core independent and dependent variables. To measure the core independent variable (internet use), we used the responses from the 2016 CFPS questionnaire to the item, “Do you use a computer at work?” (1 = use, 0 = do not use). To measure the other core independent variable (internet involvement), we used the responses to the item, “In general, how many hours do you spend online in your spare time each week?” To measure the dependent variable (cognitive health), we referred to Zhou et al. (23), the item “Can you remember the main things that happened to you in a recent week?” (1 = remember all/remember most, are classified as high cognitive health level; 0 = can remember half/can only remember a few/can only remember a little, are classified as low cognitive health level). We followed Fancourt and Steptoe (26) in measuring cognitive health in terms of word recognition ability. Finally, we adopted Li et al.'s (12) measure of cognitive health based on mean scores for memory, word recognition, and mathematical ability.

#### Changing the Sample Range

There is no consensus in the literature on the age range for middle-aged and elderly people, with some scholars including those aged 35 and above (27) and others including those aged 45 and above (28). To avoid erroneous results caused by sample selection bias, we rescreened the samples based on these definitions, selecting samples aged 35 and above and samples aged 45 and above respectively, and then carrying out robustness tests for each group.

#### Propensity Score Matching

Since the variables for internet use/involvement may be the result of individual self-selection, Order-Probit model for empirical analysis can lead to bias in the results. Therefore, to alleviate sample self-selection issues, we followed Cao et al. (29) in using propensity score matching (PSM) for robustness tests. We began with a stepwise regression method at a significance level of 10% to carry out PSM on internet use. According to the results, the matching variables finally selected for inclusion were age, years of education, political status, nonagricultural household registration, family income per capita, and exercise (4,017 samples retained). The most commonly applied PSM method, nearest neighbor matching, was then used to carry out 1:2 matching of the PS values for the internet use and nonuse groups. The matching effect satisfied the common support assumption and the balance assumption at the same time; we also used radius matching and kernel matching for further testing. The same procedure was used for internet involvement. According to the results of the stepwise regression, the matching variables finally selected were age, years of education, political status, nonagricultural household registration, family income per capita, exercise, and municipal area (12,169 samples retained).

Conducting these robustness tests yielded the results in Table 3, which show consistently that internet use positively and significantly affects the cognitive health of middle-aged and elderly people, and that internet involvement does likewise. The results obtained from the series of robustness tests are consistent with the previous results, which can therefore be regarded as having strong reliability and robustness.

**Table 3:** Results of robustness checks.

**Method**	**Endogeneity test**		**Changing the model setting**		**Changing the measuring methods**	
Internet use	0.175***		0.171***		0.166***	
	(0.035)		(0.036)		(0.032)	
Internet involvement		0.072***		0.071***		0.084***
		(0.014)		(0.014)		(0.015)
Control variable	YES	YES	YES	YES	YES	YES
City dummy	YES	YES	YES	YES	YES	YES
LR chi^2^	1834.41***	1835.00***	1843.96***	1845.12***	1117.18***	1838.20***
R^2^	0.0479	0.0480	0.0484	0.0484	0.0422	0.0481
Observations	12,130	12,128	12,085	12,083	8,412	12,126
**Method**	**Changing the measurement of cognitive health**		**Changing the sample range**			
Internet use	0.298***		0.183***			
	(0.043)		(0.035)			
Internet involvement		0.128***		0.074***		
		(0.018)		(0.014)		
Control variable	YES	YES	YES	YES		
City dummy	YES	YES	YES	YES		
LR chi^2^	1309.55***	1316.09***	1802.85***	1802.99***		
R^2^	0.0845	0.0850	0.0471	0.0471		
Observations	12,148	12,146	12,131	12,129		
**Propensity score matching**						
**Level**	**Sample**	**Test group**	**Control group**	**Difference**	**S.E**.	***T*** **-statistic**
Breadth	Unmatched	3.657	2.839	0.818	0.038	21.79
	ATT	3.657	3.442	0.215	0.060	3.57
Depth	Unmatched	3.687	2.844	0.843	0.039	21.65
	ATT	3.687	3.422	0.265	0.068	3.90

*In the robustness test that changed the measurement of cognitive health, we use word recognition ability, which includes measurements of memory, word recognition, and mathematical ability. When changing the age range of the samples, we controlled the samples for 35 years old and above and 45 years old and above, respectively. The PSM analysis method reports the results obtained using the nearest neighbor matching method, in which the results of ATU and ATE are similar to those of ATT. In addition, we used radius and kernel matching to carry out correlation analysis. Due to space limitations, we do not report all the robustness test results, which can be obtained from the authors on request.*

*** ^***^represents the significance at the 1% level.

### Heterogeneity Analysis

In the above empirical analysis, we classified all middle-aged and elderly people into a single homogeneous group. In order to explore the effects of the internet on the cognitive health of middle-aged and elderly people at different levels, we created groups based on education level (illiterate vs. literate; nine-year compulsory education or below vs. nine-year compulsory education and above; general education vs. higher education), gender (male vs. female), household registration status (nonagricultural household registration vs. agricultural household registration), location (municipality vs. non-municipality; eastern region vs. other regions; central region vs. other regions), and household composition (living with children vs. living alone).

To determine the effect of education on the relationship between the internet and the cognitive health of middle-aged and elderly people in more detail, we subdivided the middle-aged and elderly groups according to level of education. The first group of results shows that internet use (involvement) had no significant impact on the cognitive health of illiterate middle-aged and elderly people but had a significant positive impact on their cognitive health. The second group of results shows that internet use (involvement) not only had a significant positive impact on the cognitive health of middle-aged and elderly people with <9 years of compulsory education but also had a significant positive impact on the cognitive health of those with 9 years of compulsory education and above. The third group of results shows that internet use (involvement) had a significant positive impact on the cognitive health of middle-aged and elderly people with a general education but did not have a significant impact on the cognitive health of those with a higher education. In summary, the effect of the internet on the cognitive health of middle-aged and elderly people was found mainly in groups who had received a general education and not in groups who had not received an education (were illiterate) or who had received a higher education. In the fourth to seventh groups, we subdivided the samples according to household registration, gender, location, and household composition. The results show that the positive impact of internet use (involvement) on the cognitive health of middle-aged and elderly people was not significantly changed by differences in gender, household registration, location, or household composition.

To sum up, the heterogeneity analysis indicates that the impact of the internet on the cognitive health of middle-aged and elderly people is consistent across differences in gender, household registration, location, and household composition. However, there is heterogeneity in terms of level of education; that is, the effect is found only in those middle-aged and elderly groups who received a general education and not in those who received a higher education or who are illiterate. The results of heterogeneity analysis can be obtained in Table 4.

**Table 4:** Results of heterogeneity analysis.

**Group 1**	**Illiterate**	**Literate**	**Illiterate**	**Literate**
Internet use	0.134 (0.126)	0.240*** (0.037)		
Internet involvement			0.067 (0.072)	0.099*** (0.014)
LR chi^2^	363.99***	705.50***	363.71***	711.29***
R2	0.0227	0.0315	0.0226	0.0317
Observations	5,258	7,222	5,258	7,219
**Group 2**	**Below nine-year**	**Above nine-year**	**Below nine-year**	**Above nine-year**
	**compulsory education**	**compulsory education**	**compulsory education**	**compulsory education**
Internet use	0.282*** (0.046)	0.127*** (0.057)		
Internet involvement			0.140*** (0.021)	0.046** (0.020)
LR chi^2^	1,083.45***	297.20***	1,090.32***	297.36***
R^2^	0.0333	0.0465	0.0335	0.0466
Observations	10,360	2,120	10,359	2,118
**Group 3**	**General education**	**Higher education**	**General education**	**Higher education**
Internet use	0.285*** (0.037)	0.100 (0.109)		
Internet involvement			0.126*** (0.016)	0.025 (0.033)
LR chi^2^	1,488.66***	171.86***	1,493.31***	172.34***
R^2^	0.0400	0.0829	0.0401	0.0833
Observations	11,805	675	11,803	674
**Group 4**	**Nonagricultural**	**Agricultural**	**Nonagricultural**	**Agricultural**
	**household registration**	**household registration**	**household registration**	**household registration**
Internet use	0.196*** (0.047)	0.146** (0.059)		
Internet involvement			0.077*** (0.018)	0.068** (0.026)
LR chi^2^	485.46***	907.42***	486.71***	907.83***
R^2^	0.0434	0.0341	0.0436	0.0342
Observations	3,634	8,497	3,632	8,497
**Group 5**	**Male**	**Female**	**Male**	**Female**
Internet use	0.170*** (0.047)	0.179*** (0.055)		
Internet involvement			0.061*** (0.018)	0.086*** (0.022)
LR chi^2^	701.68***	867.41***	699.66***	869.83***
R^2^	0.0370	0.0457	0.0369	0.0458
Observations	6,056	6,075	6,055	6,074
**Group 6**	**Eastern area**	**Other areas**	**Eastern area**	**Other areas**
Internet use	0.171*** (0.048)	0.205*** (0.053)		
Internet involvement			0.077*** (0.020)	0.075*** (0.020)
LR chi^2^	802.85***	933.62***	804.84***	932.20***
R^2^	0.0442	0.0466	0.0444	0.0466
Observations	5,764	6,367	5,762	6,367
**Group 7**	**Living with children**	**Living alone**	**Living with children**	**Living alone**
Internet use	0.155*** (0.056)	0.185*** (0.047)		
Internet involvement			0.082*** (0.022)	0.064*** (0.019)
LR chi^2^	602.72***	1,193.86***	609.22***	1,188.16***
R^2^	0.0479	0.0479	0.0484	0.0477
Observations	3,998	7,894	3,998	7,892
Control variables	YES	YES	YES	YES

*We also analyzed whether individuals lived in the central region or in municipalities. Due to space limitations, we do not report these results, which can be obtained from the authors on request.*

*** and ** represent significance at the 1% and 5% levels, respectively.

### Further Mechanism Analysis

In the heterogeneity analysis, the results obtained by dividing the samples according to household composition indicated that the influence of the internet on the cognitive health of middle-aged and elderly people did not change significantly according to whether they lived with their children or alone. This result prompts the following considerations.

Compared to some Western countries, China is a country where family intergenerational relations are very prominent. In the context of Chinese traditional culture, the interdependent self-construal of Chinese people has led to the formation of a typical interdependent relationship between Chinese parents and their children (30). Given this context and relationship mode, Chinese parents and children show varying degrees of emotional attachment to each other at different stages of their lives. Since saving is an important means by which Chinese provide for the aged (31), and as having a child is an effective substitute for saving (32), Chinese parents who have entered into the middle-aged and elderly stage often show stronger emotional attachment to their children. In China, it is common for children to live with their parents before or after marriage. Studies have found that non-empty-nest elderly people have higher levels of mental health and better emotional status (33), whereas empty-nest elderly people experience more psychological pain, anxiety, and depression (34). This suggests that cohabitation with children is an explicit form of Chinese parents' emotional attachment to their children, and that it can satisfy that emotional attachment.

The internet has broken through the constraints of time and space. Its emergence allows parents to maintain emotional connections with children who live separately, enabling them to maintain a closer relationship and effectively satisfying their emotional attachment to their children. In the internet era, Chinese parents' emotional attachment to their children can be achieved by means other than cohabiting with them; for example, parents can use the internet as a channel for this. Studies have shown that the satisfaction of emotional attachment helps middle-aged and elderly people to stimulate positive emotions such as happiness (18), and that such positive emotions are beneficial to their cognitive health (26).

In summary, the internet helps to satisfy the emotional attachment of middle-aged and elderly people to their children, which is ultimately beneficial for their cognitive health. On this basis, we believe, first, that the satisfaction of the emotional attachment with children may be the psychological mechanism of the positive effect of the internet on the cognitive health of middle-aged and elderly people. Second, because the internet as a new tool and channel can help middle-aged and elderly people to realize their emotional attachment with their children, in the internet era (vs. the non-internet era), the effect of household composition (living with children vs. living alone) is weakened to the point that it has no significant impact on the relationship between the internet and the cognitive health of the middle-aged and elderly. Our heterogeneity analysis supports this view.

In order to determine whether the satisfaction of emotional attachment with children plays a significant mediating role in the relationship between the internet and the cognitive health of middle-aged and elderly people, we used the causal stepwise regression method proposed by Baron and Kenny (35) and the well-established bootstrap method for robustness testing (36, 37).

In line with the definition of the mediator (the satisfaction of emotional attachment with children), we used two measurements. In order to pursue the satisfaction of emotional attachment with their children, middle-aged and elderly people often increase their closeness to each other through multiple contacts. Accordingly, we used the data in the 2016 CFPS questionnaire for the item, “In the past 6 months, how often did you contact your child(ren) *via* phone calls, text messages, letters, or e-mails?” (1 = never, 2 = once every few months, 3 = once a month, 4 = two or three times a month, 5 = once or twice a week, 6 = three or four times a week, and 7 = almost every day). The responses to this item were then used as a measure of the satisfaction of emotional attachment with children.

Aspiration level theory suggests that comparison of actual income status with self-evaluated income status can accurately reflect people's material desire (23). By analogy, we suggest that comparison of the actual closeness of the relationship with the child and the self-evaluated closeness of the relationship with the child will reflect the satisfaction of middle-aged and elderly people's emotional attachment with their children. Thus we used the ratio of the scores for the actual closeness of the relationship to the scores for the self-evaluated closeness of the relationship to measure the satisfaction of middle-aged and elderly people in their emotional attachment with their children. The larger the ratio, the wider the gap between the self-evaluation and the actual closeness and the greater the satisfaction with that emotional attachment. We used the item above to measure the actual closeness of the relationship with the children. To measure the self-evaluated closeness, we used the item, “In the past 6 months, how has your relationship with your children been?” (1 = not close, 2 = not very close, 3 = normal, 4 = close, and 5 = very close).

Table 5 shows the results obtained by using the stepwise causal regression method. Internet use, as shown in column (1), had a significantly positive impact on the cognitive health of middle-aged and elderly people; according to column (2), internet use had a significantly positive impact on the satisfaction of emotional attachment with children; according to column (3), internet use significantly and positively affected the cognitive health of middle-aged and elderly people, as did the satisfaction of the emotional attachment with their children. Combining the results from columns (1) to (3) according to the principle of stepwise causal regression shows that the mediating effect of the satisfaction of emotional attachment with children was significant, and that it was an intermediate mechanism by which internet use affected the cognitive health of middle-aged and elderly people. These results are consistent with the results from columns (7) to (9). In the same way, combining the results from columns (4) to (6) shows that the mediating effect of the satisfaction of emotional attachment with children was significant, and that it was an intermediate mechanism in the relationship of internet involvement and the cognitive health of middle-aged and elderly people. These results are consistent with the results obtained in columns (10) to (12).

**Table 5:** Causal stepwise regression results for the mediating effect.

**Model**	**O-Probit**	**OLS**	**O-Probit**	**O-Probit**	**OLS**	**O-Probit**
Dependent variable	Cognitive health	1 Attachment with children	Cognitive health	Cognitive health	1 Attachment with children	Cognitive health
Column	(1)	(2)	(3)	(4)	(5)	(6)
Internet use	0.173***	0.302***	0.129**			
	(0.035)	(0.056)	(0.056)			
Internet involvement				0.071***	0.156***	0.069***
				(0.014)	(0.025)	(0.025)
Child attachment			0.042***			0.041***
			(0.006)			(0.006)
Control variable	YES	YES	YES	YES	YES	YES
City dummy	YES	YES	YES	YES	YES	YES
LR chi^2^	1,834.38***	882.26***	1,075.92***	1,834.72***	892.22***	1,078.70***
R^2^	0.0479	0.0335	0.0475	0.0480	0.0339	0.0476
Observations	12,131	7,202	7,190	12,129	7,201	7,189
**Model**	**O-Probit**	**OLS**	**O-Probit**	**O-Probit**	**OLS**	**O-Probit**
Dependent variable	Cognitive health	2 Attachment with children	Cognitive health	Cognitive health	2 Attachment with children	Cognitive health
Column	(7)	(8)	(9)	(10)	(11)	(12)
Internet use	0.173***	0.091***	0.138**			
	(0.035)	(0.026)	(0.056)			
Internet involvement				0.071***	0.048***	0.074***
				(0.014)	(0.011)	(0.025)
Child attachment 2			0.122***			0.121***
			(0.025)			(0.025)
Control variable	YES	YES	YES	YES	YES	YES
City dummy	YES	YES	YES	YES	YES	YES
LR chi^2^	1,834.38***		1,055.80***	1,834.72***		1,058.85***
F		40.16***			40.38***	
R^2^	0.0479	0.0821	0.0466	0.0480	0.0825	0.0467
Observations	12,131	7,202	7,190	12,129	7,201	7,189

*“1 Attachment with children” represents the mediator variable measured using the first method; “2 Attachment with children” represents the mediator variable measured using the second method. Due to space limitations, the results of other variables have not been reported and can be obtained from the authors on request.*

*** and ** represent significance at the 1% and 5% levels, respectively.

Table 6 gives the results obtained by using the “*Bootstrap*” method on a sample of 5,000 cases and choosing Model (4) at a 95% level of confidence. The results consistently show that the mediating effect (the satisfaction of the emotional attachment with children) exists in the relationship between internet use and the cognitive health of middle-aged and elderly people (*LLCI* = 0.102, *ULCI* = 0.154; *LLCI* = 0.064, *ULCI* = 0.104); the interval does not contain 0, and the mediating effect is a partial mediator. Moreover, the mediating effect also exists in the effect of internet involvement on the cognitive health of middle-aged and elderly people (*LLCI* = 0.044, *ULCI* = 0.067; *LLCI* = 0.027, *ULCI* = 0.045); again, the interval does not contain 0, and the mediating effect is a partial mediator. These results are consistent with the results obtained using the stepwise causal regression method, which indicates that the satisfaction of emotional attachment with children has a robust mediating effect on the impact of the internet on the cognitive health of middle-aged and elderly people.

**Table 6:** Bootstrap analysis results for the mediating effect.

		**LLCI**	**ULCI**	**LLCI**	**ULCI**
1 Attachment with children	Indirect effect	0.102	0.154	0.044	0.067
	Direct effect	0.673	0.879	0.278	0.367
2 Attachment with children	Indirect effect	0.064	0.104	0.027	0.045
	Direct effect	0.718	0.923	0.298	0.386

“*1 Attachment with children” represents the mediator variable measured using the first method; “2 Attachment with children” represents the mediator variable measured using the second method*.

## Discussion and Conclusions

**Figure 3:**
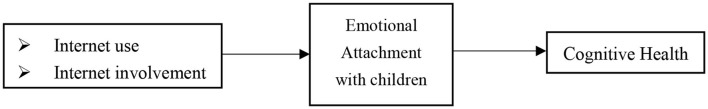
The framework.

To promote the construction of a healthy China, our paper is dedicated to discovering pathways that can improve the cognitive health of middle-aged and elderly people. Through in-depth analysis, as Figure 3. suggests, we find that the internet has a significant positive impact, as internet use and involvement significantly and positively affect the cognitive health of people in that age group. These positive effects do not change significantly with differences in gender, household registration, location, or household composition, but they are heterogeneous in relation to levels of education; that is, the effect exists only in those who received a general education and not in those who are illiterate or who received a higher education. Through further mechanism analysis, we find that the satisfaction of emotional attachment with children is the psychological mechanism of the internet's influence on the cognitive health of middle-aged and elderly people.

Based on the above conclusions, we propose the following measures to realize a healthy China strategy. First, to maintain and improve the cognitive health of middle-aged and elderly people, the Chinese government can use the internet as a tool. It is worth noting that the internet makes demands on the cognition and technological skills of users, and middle-aged and elderly people may have limitations in these respects. Accordingly, the government can increase investment and services to help middle-aged and elderly people learn to use the internet. Similarly, young people should actively and patiently help their parents integrate into the internet era so that they will not be left behind. Companies, also, can produce internet devices that are designed specifically for use by middle-aged and elderly people. Second, children living separately from their parents should communicate with them through the internet frequently to satisfy their parents' emotional attachment and improve their cognitive health. Third, the government should continue to invest in education to alleviate the deterioration of the cognitive health of middle-aged and elderly people in the internet era. Nevertheless, it should be noted that for middle-aged and elderly people who have had a higher education, other types of mitigation need to be explored.

This study has a number of limitations, and these represent research directions that can be explored further. First, the measurement of cognitive health of middle-aged and elderly people is limited. Since the data accessibility, in the basic model, we only use one single item (question) to measure the cognitive health of middle-aged and elderly people. However, the cognitive health might be a quite complex dimension of health, using a single item to measure may not be the most perfect method. In the future analysis, we will try to find more related items to measure the cognitive health, to better ensure the reliability and validity of the research.

Second, it is reasonable to assume that emotions are another potential mechanism by which the internet affects the cognitive health of middle-aged and elderly people. Although previous studies have confirmed that the internet has a general effect on health through the emotions (17, 21), emotion as a mediator in the relationship between the internet and the cognitive health of middle-aged and elderly people has been analyzed a lot and played a significant or substantial theoretical role. Since the aim of our study is to reveal the psychological mechanism in the Chinese cultural context, and given the above considerations and the limitations of the research data, we have not verified emotion as a possible underlying mechanism. Instead, drawing on a functional perspective on the internet, we have explored its impact on the cognitive health of middle-aged and elderly people and the mechanism of that impact.

Third, in addition to its functional value, the internet today may operate as an invisible social label; that is, the internet has social functions. Because of limitations of space, we have not considered this point here. However, it can be noted that young people are the main users of the internet, and that middle-aged and elderly people have become marginalized in the internet era because of internal or external factors. The emergence of the internet has thus created a “digital gap” between the young and middle-aged and elderly groups (38). Facing this gap, young people may assume that middle-aged and elderly people do not understand the internet and cannot integrate into the internet era. This may lead them to classify middle-aged and elderly people as unable to keep up with the times (in short, as “Out”). In essence, this is a stigmatization of middle-aged and elderly people, which may cause them to experience deprivation and have a negative impact on their health. In future, therefore, researchers can enhance the theoretical and practical value of the present study by exploring the effect of the internet on the health of middle-aged and elderly people from the perspective of its social function.

## Data Availability Statement

The original contributions presented in the study are included in the article/supplementary material, further inquiries can be directed to the corresponding author/s.

## Ethics Statement

The studies involving human participants were reviewed and approved by China National Knowledge Infrastructure (CNKI) Ethics Committee. Written informed consent to participate in this study was provided by the participants' or participants' legal guardian/next of kin.

## Author Contributions

All authors listed have made a substantial, direct, and intellectual contribution to the work and approved it for publication.

## Funding

This paper was supported in part by the National Social Science Foundation of China (No. 21CGL019).

## Conflict of Interest

The authors declare that the research was conducted in the absence of any commercial or financial relationships that could be construed as a potential conflict of interest.

## Publisher's Note

All claims expressed in this article are solely those of the authors and do not necessarily represent those of their affiliated organizations, or those of the publisher, the editors and the reviewers. Any product that may be evaluated in this article, or claim that may be made by its manufacturer, is not guaranteed or endorsed by the publisher.

## Footnotes

^1^These data come from *Blue Book of Elderly Health: China's Elderly Health Research Report* (2018). Available online at: https://baijiahao.baidu.com/s?id=1623865115324600959&wfr=spider&for=pc

^2^The data come from the Guanghua Best Consumer Big Data Center, and are available online at: http://www.bestmarketing.cn/

^3^The data come from the 40th Statistical Report on the Development of China's Internet. Available online at: http://media.people.com.cn/GB/179618/413695/index.html

^4^This is the only year that contains the complete data required for our study.

^5^Details please see http://www.isss.pku.edu.cn/cfps/en/index.htm?CSRFT=9GPI-97MW-5FGN-REG1-YFMZ-TMJ0-3Q8J-1T44
